# Metabolomics as a potential tool for monitoring patients with aneurysmal subarachnoid hemorrhage

**DOI:** 10.3389/fneur.2022.1101524

**Published:** 2023-01-09

**Authors:** Nebojsa Lasica, Vidak Raicevic, Nikola M. Stojanovic, Djula Djilvesi, Igor Horvat, Bojan Jelaca, Filip Pajicic, Petar Vulekovic

**Affiliations:** ^1^Faculty of Medicine, University of Novi Sad, Novi Sad, Serbia; ^2^Clinic of Neurosurgery, University Clinical Center of Vojvodina, Novi Sad, Serbia; ^3^Department of Chemistry, Biochemistry and Environmental Protection, Faculty of Sciences, University of Novi Sad, Novi Sad, Serbia; ^4^Department of Physiology, Faculty of Medicine, University of Niš, Niš, Serbia

**Keywords:** metabolomics, metabonomics, stroke, subarachnoid hemorrhage, intracranial aneurysm, neurocritical care

## Abstract

Metabolomics has evolved into a particularly useful tool to study interactions between metabolites and serves as an aid in unraveling the complexity of entire metabolomes. Nonetheless, it is increasingly viewed as a methodology with practical applications in the clinical setting, where identifying and quantifying biomarkers of interest could prove useful for diagnostics. Starting from a concise overview of the most prominent analytical techniques employed in metabolomics, herein we present a review of its application in studies of brain metabolism and cerebrovascular diseases, paying most attention to its uses in researching aneurysmal subarachnoid hemorrhage. Both animal models and human studies are considered, and metabolites identified as potential biomarkers are highlighted.

## 1. Background

Systems biology strategies to study relationships between biomolecules and biological processes in the past relied on the analysis of particular biomolecules and related pathways, with no information considering entire biological systems. The need for a complete overview of a specific set of biomolecules at any level of organization (e.g., DNA, RNA, protein, lipid, metabolite) led to the development of the omics approaches that generated an immense amount of data from biofluids, cells, tissues, or organisms. The term metabolomics was first coined by Oliver et al. (1); they defined it as the comprehensive and quantitative analysis of all metabolites of the biological system under study (2). In 1999 Nicholson et al. used the term metabonomics as 'the quantitative measurement of the dynamic multiparametric metabolic response of living systems to pathophysiological stimuli or genetic modification' (3). Due to similarities in definition, both terms are used interchangeably in the literature, with metabolomics being used more commonly.

Metabolomics relies on using analytical chemistry techniques that allow profiling and characterization of molecules in the specimen that provides data that ultimately leads to the identification of metabolites and metabolic pathways (4). This enabled metabolomics to be at the forefront of biomarkers research and helped understand subtle changes in biological systems in both physiological conditions and disease (5). As concentrations, polarities, and molecular masses of metabolites are quite diverse, the inherent problem of metabolomics is that no single analytical tool is sufficient and sensitive enough to measure the span of chemical entities present in the sample. Following the completion of the Human Genome Project, most of the human genome and corresponding transcriptome and proteome were known, and the database was made electronically available (6). On the other hand, a comprehensive investigation of the human metabolome was still lacking at that time. To address this, the Human Metabolome Project was launched in 2004 aiming at identifying and quantifying detectable molecules (>1 mM) in several bodily fluids, and made data from an electronic database called the Human Metabolome Database (HMDB) publicly available, rendering it the most comprehensive organism-specific database to date (7).

In the present work, we discuss how metabolomics has been used to study brain metabolism and cerebrovascular diseases with a particular reference to aneurysmal subarachnoid hemorrhage (aSAH). We briefly describe the most widely used tools in metabolomics research and overview their application in studying cerebrovascular diseases, with particular emphasis on potential biomarker identification in patient groups.

## 2. Tools to study metabolomics

For the purposes of research based on metabolomics, it is necessary to use such an analytical technique that would enable simultaneous detection and quantification of numerous metabolites, often in demanding biological matrices. Unfortunately, due to the complexity of metabolomes, which include a plethora of compounds that differ by physicochemical properties and concentration, a single analytical technique can never adequately address all metabolites in a given sample, which renders it a necessity to employ a multiplatform analysis if an extensive investigation is to be carried out to maximize metabolome coverage (8, 9). A comparison of some of the most widely employed techniques in metabolomics is presented in Supplementary Table 1.

**Figure 1:**
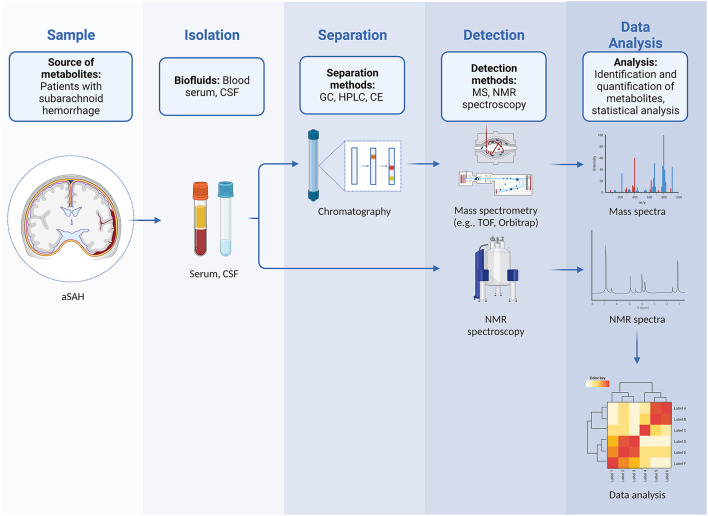
Workflow in untargeted metabolomics in patients with aSAH showing steps that include sampling and isolation of biological fluids, separation, detection of metabolites, and subsequent data analysis with identification and quantification of metabolites.

Any metabolomics experiment can either be targeted or untargeted. Targeted studies focus on quantifying a small number of metabolites (which are frequently chemically related), and usually involve extensive sample preparation procedures to optimize detection and quantification. Conversely, untargeted studies (also referred to as metabolic profiling) strive to achieve the detection of a wide range of metabolites and often result in finding new or hitherto unexpected compounds (Figure 1). Most platforms currently used in metabolomics are based either on mass spectrometry (MS) or nuclear magnetic resonance (NMR) spectroscopy (8).

MS is based on the formation of charged species which are subsequently separated by their mass-charge ratio (*m*/*z*) (8). As most metabolite-derived species are singularly charged, mass spectrometry essentially provides information regarding molecular weights, but specificities in each platform setup provide additional clues which lead to analyte identification, often with the aid of spectral libraries and/or chromatographic parameters. Mass spectrometry is usually performed after chromatographic separation, which is the basis of the so-called hyphenated techniques, GC-MS and LC-MS (10). Metabolites (or their derivatives) that can vaporize when exposed to temperatures under 350°C can be subjected to gas chromatographic (GC) separation (8, 11). Liquid chromatographic (LC) separation is more versatile, but is generally thought of as providing lower chromatographic separation than GC, and is most adequate for non-polar metabolites (8, 12).

When applied in metabolomics, NMR is in a majority of cases directed at observing different hydrogen nuclei, which produce signals of specific chemical shifts and fine structure depending on their chemical environment (8, 9, 12). As biofluids contain a significant amount of water, the signal of the water hydrogen nuclei must be suppressed, or valuable data about metabolite hydrogen nuclei might be lost. Signals arising from nuclei of large molecular weight molecules can also be attenuated. Other spin-active nuclei such as ^13^C and ^31^P can also be studied. In the form of magnetic resonance imaging (MRI), a three-dimensional spatial mapping of metabolites is also possible (8).

## 3. Stroke metabolomics

Alterations in cellular metabolism preceding and during stroke induce local and systemic metabolic changes that sparked interest in investigating potential biomarkers. Such alterations may reflect the downstream function of a gene, environmental changes, and also changes in cellular signaling. Therefore, capturing the profile of circulating metabolites may provide systems-level information and insight into particular disease mechanisms, but also predict the occurrence of index events, differentiate the substrata of stroke patients, and predict the functional outcome (13).

### 3.1. Animal models of stroke and metabolic profile

During brain damage, the changes in the metabolic profile could occur both in the brain tissue and in plasma (blood), and choosing to examine only one of the two samples could lead to changes being overlooked, since the dysregulation in one bodily system does not mean the same would follow in another (14). However, in animal studies, the tissue samples are readily available, while in human-based studies researchers mainly depend on a blood sample and, in some cases, cerebrospinal fluid (CSF). Animal studies also enable a better understanding of how and when dysregulation occurs; e.g., blood samples can be obtained before, during, and after the animal experiments, providing further insight into metabolic pathways.

**Figure 2:**
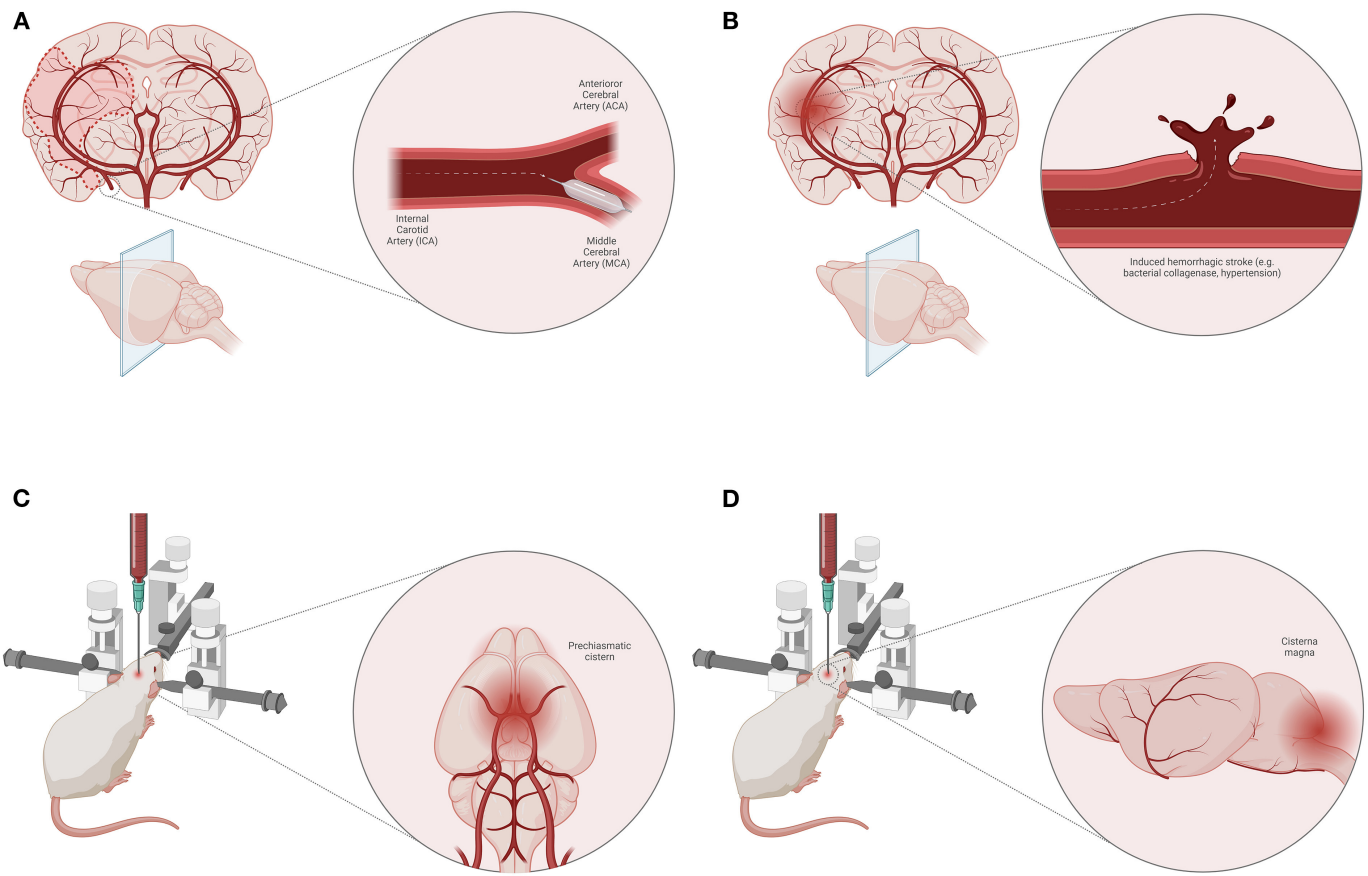
Animal models of stroke. Middle cerebral artery (MCA) occlusion model of ischemic stroke **(A)**. Induced hemorrhagic stroke animal model (e.g., bacterial collagenase, induced hypertension) **(B)**. Blood injection techniques for SAH induction—pre-chiasmatic cistern **(C)** and cisterna magna **(D)** model.

The animal models of ischemic stroke are an invaluable tool for exploring the pathophysiological mechanisms of ischemia/reperfusion (14). They have numerous advantages, such as high reproducibility, control over the ischemic area, and standardization of the experimental protocol which result in consistent conclusions regarding changes occurring at the molecular level. The most frequently used model for ischemic stroke is middle cerebral artery occlusion (Figure 2A), however, this model is not without its limitations and only moderately mimics the pathophysiology of ischemic stroke seen in humans (14). Mainly, amino acid levels (e.g., glycine and lysine) are increased both in the brain and plasma of animals 2 days after stroke, however, the levels of methionine are only increased in brain tissue, while at the same time there is a decrease in plasma concertation of this amino acid in animals with stroke (15). In certain cases, the results from animal studies are not fully in line with those of human studies. For example, lysophosphatidylcholine, partially related with different oxidative cell damage and inflammation induction by a hitherto unknown mechanism, is found to be associated with stroke occurrence and recurrence in humans, while the same role was not observed in animals (16). However, the studies from animals and humans generally overlap and one could reach the same conclusions from the results. This is in particular true for alanine, aspartate, and glutamate metabolism which are found to be associated with a high risk for ischemic stroke in both animals and humans (17). Apart from the metabolic profile, there is a dysregulation in the cell energy which could easily be monitored through the levels of ADP, uric acid, and lactates (17).

Spontaneous hemorrhagic stroke occurs rarely in disease-free animals, however, hemorrhagic stroke can be induced by injecting bacterial collagenase or blood into the brain parenchyma (18) (Figure 2B). This model could also be applied on spontaneously hypertensive animals (18). Although these models have some advantages, such as precise induction, and controlled volume of injecting agent, there are other disadvantages related to animals (e.g., their health status) and therapeutic approaches. In some cases, hemorrhagic stroke may be associated with poor polyamine metabolism (in genetically mutated polyamine-modulated factor 1) which results in altered blood and CSF spermine, spermidine, and putrescine levels (19). High levels of plasma homocysteine are known to cause endothelial dysfunction associated with atherosclerosis which makes it a risk factor for hemorrhagic stroke (19). Above all, one of the main risk factors for atherosclerosis and associated hemorrhagic stroke is a disturbance in lipid profile, i.e., high HDL and cholesterol, and low LDL plasma levels (19). Regarding energy depletion in animals with hemorrhagic stroke, an increase in levels of tissue serine, a ketogenic and glucogenic amino acid, is a signal of cell starvation (20). Nonetheless, an increase in levels of phenylalanine and L-lysine could indicate a poor prognosis since they are known to increase glutamate neuron firing (21).

In animal models, subarachnoid hemorrhage (SAH) is quite hard to simulate so that it could correspond to the SAH occurring in humans. The main cause of SAH in humans is the rupture of an aneurysm, while the rodent models of SAH are defined as non-aneurysmal (22). In an attempt to do so, several techniques are performed, such as injection of blood into the subarachnoid space/cerebral ventricles or by endovascular perforation of a subarachnoid vessel. It is believed that an adequate method that models the pathophysiology in humans most closely is the intraluminal Circle of Willis perforation injury at the internal carotid artery bifurcation (23). Nonetheless, other models such as blood (homologous or heterologous) injection into pre-chiasmatic cistern (Figure 2C) and cisterna magna (Figure 2D) compromising anterior and posterior blood supply, respectively, are frequently used (22). Apart from the standard physiological parameters affected by SAH, which could be tracked in animal experiments (e.g., blood pressure, oxygen tension, cerebral perfusion pressure, etc.), contemporary studies are focused on finding specific metabolic products for SAH (22).

One of the well-known molecules associated with a more unfavorable prognosis of SAH is hemoglobin, and its increased content in CSF is found to be in correlation with cerebral ischemia and poor neurological outcome of affected animals (24). Vasospasm, a frequent complication seen in SAH patients is known to be directly related to increased concentrations of hemoglobin in CSF, as well as with bilirubin oxidation end products (BOXes), propentdyopents and Fe^3+^ ions (25). The products of energy-supplying molecules are significantly disturbed in the cerebrospinal fluid (CSF), indicating the suffering of nerve and glial cells. Often there is an increase in citrate, lactate, and pyruvate, an increased concentration of amino acids, and intensified lipid metabolism (26).

### 3.2. Human studies and metabolic profiles in stroke patients

#### 3.2.1. Metabolites and stroke risk

Risk stratification prior to the index event would enable advances in prevention and therapy in patients with a particular propensity toward the development of stroke, regardless of etiology. Early metabolomic studies in elderly patients, however, limited by the number of participants, were able to identify medium- and long-chain acylcarnitines together with alanine as independent predictors of major cardiovascular events (MACE), including stroke (27). Follow-up studies on larger samples further identified a correlation between plasma ceramides and phenylacetylglutamine, a gut microbiota-derived metabolite, and increased risk through altered platelet activation and responsiveness (28, 29). Lower levels of lysine catabolites and a group of metabolites, most of them amino acids, using both targeted and untargeted approaches were found to be potential markers of thrombotic stroke (30, 31). In contrast, direct and inverse relationships between hexadecanedioate and sphingomyelin were found in ischaemic stroke, respectively (32, 33). However, one study found that none of the metabolites was consistently associated with the risk for stroke (34).

#### 3.2.2. Metabolic signature in stroke patients

Timely diagnosis and evaluation of patients with acute stroke are essential for prompt treatment. Still, in the clinical setting, imaging modalities used for diagnosis may not reveal any changes early on and may be time-consuming, demonstrating the need for novel and sensitive biomarkers for early diagnosis. Thus far, studies were able to identify metabolomic biomarkers of transient ischemic attack (TIA) (35), ischemic (26, 36) and hemorrhagic stroke (37, 38), metabolites capable of distinguishing between the stroke types (39–41) and stages (42), but also to differentiate between stroke and stroke mimics (43). It is evident that there is strong diagnostic potential from discovering differential biomarkers. Even so, there are no routinely used metabolic biomarkers employed in the clinical setting to date.

#### 3.2.3. Metabolomics of aneurysmal SAH and future perspectives

Despite extensive research, predictors of outcome in patients with aSAH remain poorly understood. However, there has been a recent effort to utilize novel machine-learning methods to substantiate metabolomic biomarkers, both from blood plasma samples and the CSF, which could be potentially used in the clinical setting to foretell a functional outcome in patients with aSAH. Plasma concentrations of taurine and CSF levels of symmetric dimethylarginine, dimethylguanidino valerate, and ornithine were able to predict the 90-day functional outcome measured by the modified Rankin Scale (44, 45). Another study investigating the CSF metabolome of aSAH patients revealed differences including carbohydrate, lipid, and protein metabolism when compared to control subjects. In addition, it revealed a correlation between the pyruvate concentration and its metabolism and the severity of SAH (46, 47). Despite recent interest in providing novel insights into potential biomarkers in patients with aSAH, many questions concerning the stratification and detection of differences in patients that develop delayed cerebral ischemia, vasospasm, and other possible complications during the hospital stay and after discharge still remain unanswered, and open for future investigation.

## 4. Conclusion and future perspectives

There was an extensive prior effort to define metabolic profiles of stroke in both animal models and patients that may be used in the clinical setting. A recent shift of interest toward potential applications in aSAH patients, in particular, in neurocritical care, which includes risk stratification of patients and emphasis on predicting the development of early complications after the aneurysm rupture, may provide invaluable monitoring tools that will ensure better care for aSAH patients. Technological advances and increasing affordability of analytical equipment will play a pivotal role in the future implementation of omic tools, including metabolomics, not only for research purposes but for everyday patient care.

## Author contributions

NL, VR, and NS reviewed literature and wrote the manuscript. NL created illustrations. VR compiled the supplementary table. All authors contributed to the critical revision of the drafted article and approved the submitted version.
