# Supplementary material for: Metabolomics as a potential tool for monitoring patients with aneurysmal subarachnoid hemorrhage
Source: Front Neurol. 2023 Jan 9;13:1101524. doi: 10.3389/fneur.2022.1101524 (PMC9868237; doi:10.3389/fneur.2022.1101524)
Supplement: Supplementary file 1 [file Table_1.DOCX]

Supplementary Table 1. An overview of the most frequently encountered analytical techniques in metabolomics

| Analytical technique | Type of detectable metabolite | Advantages | Disadvantages |
| --- | --- | --- | --- |
| GC-MS | Volatile metabolites (and some nonvolatile metabolites after derivatization) of various polarities | - High sensitivity - Sample preparation is straightforward - More robust than LC-MS - Metabolite identification can be performed without a reference compound sample | - Limited ability to detect nonvolatile analytes - Determination of response factors necessary for quantification |
| LC-MS | Volatile or nonvolatile primarily nonpolar metabolites | - High sensitivity - Does not require derivatization of nonvolatile metabolites | - Determination of response factors necessary for quantification - Periodic quality assurance needed - Reference compound samples needed |
| NMR | All metabolites | - Non-destructive - Minimal sample preparation - Highly reproducible - Quantitative (^1^H) - Spatial mapping possible (MRI) | - Sensitivity inferior to MS - Metabolite identification hindered by signal overlap issues |
